# Major alterations to monocyte and dendritic cell subsets lasting more than 6 months after hospitalization for COVID-19

**DOI:** 10.3389/fimmu.2022.1082912

**Published:** 2023-01-04

**Authors:** Francis R. Hopkins, Melissa Govender, Cecilia Svanberg, Johan Nordgren, Hjalmar Waller, Åsa Nilsdotter-Augustinsson, Anna J. Henningsson, Marie Hagbom, Johanna Sjöwall, Sofia Nyström, Marie Larsson

**Affiliations:** ^1^Division of Molecular Medicine and Virology, Department of Biomedical and Clinical Sciences, Linköping University, Linköping, Sweden; ^2^Division of Infection and Inflammation, Department of Biomedical and Clinical Sciences, Linköping University, Linköping, Sweden; ^3^Department of Infectious Diseases, Linköping University, Linköping, Sweden; ^4^Division of Clinical Microbiology, Department of Laboratory Medicine in Jönköping, Ryhov County Hospital, Jönköping, Sweden; ^5^Department of Clinical Immunology and Transfusion Medicine, Linköping University, Linköping, Sweden

**Keywords:** SARS-CoV-2, COVID-19, myeloid compartment, dendritic cells, monocytes

## Abstract

**Introduction:**

After more than two years the Coronavirus disease-19 (COVID-19) pandemic continues to burden healthcare systems and economies worldwide, and it is evident that the effects on the immune system can persist for months post-infection. The activity of myeloid cells such as monocytes and dendritic cells (DC) is essential for correct mobilization of the innate and adaptive responses to a pathogen. Impaired levels and responses of monocytes and DC to severe acute respiratory syndrome coronavirus 2 (SARS-CoV-2) is likely to be a driving force behind the immune dysregulation that characterizes severe COVID-19.

**Methods:**

Here, we followed a cohort of COVID-19 patients hospitalized during the early waves of the pandemic for 6-7 months. The levels and phenotypes of circulating monocyte and DC subsets were assessed to determine both the early and long-term effects of the SARS-CoV-2 infection.

**Results:**

We found increased monocyte levels that persisted for 6-7 months, mostly attributed to elevated levels of classical monocytes. Myeloid derived suppressor cells were also elevated over this period. While most DC subsets recovered from an initial decrease, we found elevated levels of cDC2/cDC3 at the 6-7 month timepoint. Analysis of functional markers on monocytes and DC revealed sustained reduction in program death ligand 1 (PD-L1) expression but increased CD86 expression across almost all cell types examined. Finally, C-reactive protein (CRP) correlated positively to the levels of intermediate monocytes and negatively to the recovery of DC subsets.

**Conclusion:**

By exploring the myeloid compartments, we show here that alterations in the immune landscape remain more than 6 months after severe COVID-19, which could be indicative of ongoing healing and/or persistence of viral antigens.

## Introduction

Since coronavirus disease 2019 (COVID-19) was declared a pandemic on March 11, 2020, great progress has been made in combating the disease. To date, more than ten vaccines have been developed and granted emergency use listing by the World Health Organization (1). As of October 2022, the mortality from the disease stands at 6.5 million deaths, with 626 million cumulative cases worldwide (2), although with different standards of testing worldwide, the true human cost of the pandemic is likely to be much higher. The evolution of new variants indicate that the pandemic is still far from over.

The innate and adaptive immune responses during severe acute respiratory syndrome coronavirus 2 (SARS-CoV-2) infection have been extensively studied. Different pathways, markers, and factors of interest for drug targets or vaccine development have been identified and provide a better understanding of the impact of COVID-19 on the human host. While the various T cell responses in COVID-19 have been delineated (3–7), there is still much unknown about other immune cells and their role in the disease.

The myeloid cell compartment consists of a variety of innate immune cell populations including dendritic cells (DC) and monocytes (8). Monocytes can be divided into different subsets based on their expression levels of CD14 and CD16 (9). Classical monocytes (cMo, CD14^+^CD16^-^) are highly phagocytic and can upregulate proteins associated with anti-bacterial activity, while non-classical monocytes (ncMo, CD14^-/low^CD16^+^) have a less inflammatory phenotype and are associated with wound healing (10). Intermediate monocytes (iMo, CD14^+^CD16^+^) are not completely understood but display a capacity for both antigen presentation and inflammatory responses (11, 12). In addition to monocytes, there are also myeloid derived suppressor cells (MDSC) that are strongly immunosuppressive, which increase in numbers in settings such as chronic infections and cancers (13).

The pathogenic role of monocytes in respiratory viral infection has been demonstrated in mouse models of influenza A infection, where inflammatory monocytes drive the virus-induced damage to the lung (14). Alterations to the monocyte compartment occur during SARS-CoV-2 infection, with most findings showing strong inflammatory monocyte responses in severe disease (15, 16), whereas the opposite is found for mild disease (17). Indeed, it has been shown that classical monocytes are drivers of the cytokine storm that frequently proves fatal in SARS-CoV-2 infection (18).

The DC serve as a bridge between the innate and adaptive responses and are essential for the development of an adaptive response (19). Like monocytes, DC can be classified into different subsets with the ability to sense pathogens, produce cytokines and activate T cell responses (20). Plasmacytoid DC (pDC) are defined by surface expression of CD303 (BDCA-2) and are the main type I interferon (IFN) producers (21, 22). Conventional DC (cDC) are further divided into different subsets, with cDC1 having a high capacity to cross-present antigens and activate T helper (Th)1 responses (20, 23), while cDC2 activate a wider range of Th responses (20). Recent work has divided cDC2 further, based on their CD5 expression, into a CD5^high^ population mainly promoting Th1 responses, and a CD5^low^ population promoting Th2, Th17, Th22, and T regulatory cell responses (24). In addition, within the traditionally defined cDC2 population the most newly characterized populations are the two cDC3 populations (20).

Functionality of monocyte and DC subsets can be defined by their expression of surface markers such as HLA-DR, CD86, CCR7 and PD-L1. In COVID-19 patients there is evidence of lower HLA-DR expression on DC subtypes, but not monocytes, indicating an impaired ability to activate T cells. Increased PD-L1 expression is also indicative of an impairment of the effector function of DC (25).

Studies of mild and severe COVID-19 patients have demonstrated an early overall decrease in circulating DC populations, particularly the pDC (26, 27). Furthermore, in severe disease the different DC subsets are impaired in their ability to sense pathogens, present antigens, and stimulate T cell responses (4, 28, 29). The importance of a functional DC compartment is illustrated by the correlation between the activation of early and effective T cell responses, and a positive clinical outcome in SARS-CoV-2 infection (30, 31). So far, there have been few longitudinal studies investigating the effects of COVID-19 on myeloid cell subsets during the disease and after recovery (32), and more data is needed to provide better insight into the long-term effects of COVID-19.

Here, we have characterized the effects that severe COVID-19 exerts on circulating DC, monocytes, and MDSC, in patients that needed hospitalization, using spectral flow cytometry. Furthermore, the effects of several clinical markers on levels of myeloid cell subsets were assessed.

In accordance with previous studies, all circulating DC subsets were decreased at inclusion, during acute infection. While pDC and cDC1 subsequently returned to levels comparable to healthy controls, the cDC2 and cDC3 combined subset was significantly elevated at the 6-7 month time point. We further found the frequencies of different monocyte subsets to be altered. An initial increase of cMo and decrease of iMo and ncMo was observed, and this profile was maintained long-term for cMo and iMo in comparison to healthy controls. Additionally, the MDSC compartment remained elevated, confirming a long-term immunosuppressive environment post-COVID-19. In addition, there was an alteration of immunomodulatory markers, such as HLA-DR, CD86 and PD-L1, in all cell subsets. Together, our findings highlight sustained long-term alterations in monocyte and DC subsets after COVID-19, which could be linked to initial elevated circulating C-reactive protein (CRP) levels.

## Materials and methods

### Clinical data and study design

This study was approved by the Swedish Ethical Review Authority (Ethics No. 2020-02580). The hospitalized COVID-19 patients were included at the Clinic of Infectious Diseases and the Intensive Care Unit (ICU) at the Vrinnevi Hospital, Norrköping, Sweden. Furthermore, healthy COVID-19 negative controls were enrolled among the staff at the same hospital. For this study we used longitudinal samples from hospitalized COVID-19 patients (N=21; age range 32-83 years) and samples from healthy controls (N=16; age range 23-80 years). Sample collection from COVID-19 patients was performed at four timepoints throughout the study: at enrolment when the COVID-19 patients were admitted to the Department of Infectious Diseases and after 2 weeks, 6 weeks, and 6-7 months post-enrolment. Both male and female participants were included, and all individuals had provided written informed consent prior to enrolment. This study was carried out in accordance with the International Conference on Harmonization Guidelines and the Declaration of Helsinki. Clinical data is described in **Table 1**.

**Table 1:** Clinical and-demographical characteristics of hospitalized COVID-19 patients.

Variable	Clinical data	Reference range
**Number of patients**	21	
**Age, median (range)**	56 (32–83)	
**Body mass index, median (range)**	30.7 (23.7–45.2)	
**Biological sex, % (N)**	38.1 F/61.9 M (8F/13M)	
**Days in hospital, median (range)**	9 (4-34)	
**ICU/pandemic Ward %, (N)**	14.3/85.7 (3/18)	
**Days with symptoms before inclusion, median (range)**	11 (5-30)	
**Spike IgG antibody positive at inclusion, % (N)**	76.2 (16)	
**Nucleocapsid IgG antibody positive at inclusion, % (N)**	76.2 (16)	
**Viral load at inclusion, median (range)(copies/ml)**	3589 (1071-111x10^6^)	
**Antiviral treatment, % (N)**	42.9 (9)	
**Corticosteroid treatment, % (N)**	76.2 (16)	
**No/oxygen/HFNOT : CPAP^1^/mechanical ventilation, % (N)**	4.8/38.1/52.3/4.8 (1/8/11/1)	
**Cardiovascular disease, % (N)**	52.4 (11)	
**Pulmonary disease, % (N)**	28.6 (6)	
**Diabetes mellitus, % (N)**	19 (4)	
**Two of the underlying conditions, % (N)**	19 (4)	
**Three of the underlying conditions, % (N)**	9.5 (2)	
**Disease score: moderate/severe, % (N)**	85.7 (18)/14.3 (3)	
**Smoker/snus, % (N)**	19 (4)	
**Previous history of smoking/snus, % (N)**	47.6 (10)	
**Leukocytes (x 10^9^/L), median (range)**	6.6 (3.5–20.4)	3.5–8.8
**Thrombocytes (x 10^9^/L), median (range)**	275 (151–476)	150–400
**Lymphocytes (x 10^9^/L), median (range)**	0.9 (0.2–2.8)	1.1–4.8
**Monocytes (x 10^9^/L), median (range)**	0.4 (0.1-1.33)	0.1–1
**Lactate dehydrogenase (µKat/L), median (range)**	7.4 (3.9–16)	>70 years <3.5, <70 years < 4.3
**C-reactive protein (mg/L), median (range)**	86 (14–295)	0–10

^1^High flow nasal oxygen therapy (HFNOT), continuous positive airway pressure therapy (CPAP).

Diabetes mellitus, cardiovascular, and chronic pulmonary diseases defined by the individuals being medicated for these conditions.

Data regarding the clinical symptoms related to COVID-19 and general health status were collected from all participants included in the study and laboratory blood analyses of an array of factors such as CRP and LDH, were performed at the Clinical Chemistry department at Linköping University Hospital. The information is summarized in **Table 1**. The severity of the disease was determined in the individuals hospitalized for COVID-19 as per the criteria defined by the National Institutes of Health (33), i.e., estimated with regards to maximum oxygen needed, and highest level of care provided. COVID-19 severity was classified as: mild (admitted to pandemic department, no oxygen supplementation), moderate (admitted to pandemic department, oxygen supplementation <5L/min), severe (admitted to pandemic department or intermediate care unit, oxygen need ≥5L/min, supplemented by high flow nasal oxygen therapy (HFNOT) and continuous positive airway pressure therapy (CPAP)) and critical (intensive care unit, with or without mechanical ventilation). In addition, some 6-7 month samples affected by vaccination against COVID-19 were excluded from the analysis.

### Sample collection and processing

Blood samples were collected from study subjects in EDTA-treated Vacuette^®^ tubes (Griener Bio-one GmbH, Kremsmünster, Austria) and peripheral blood mononuclear cells (PBMCs) were isolated by density gradient centrifugation (Ficoll-Paque, GE Healthcare, ThermoFisher). The PBMCs were washed and cryopreserved in freezing medium (8% DMSO in FBS) at -150°C until use. Sera were isolated from whole blood samples that had been collected in 3.5 ml Vacuette^®^ tubes (Griener Bio-one GmbH, Kremsmünster, Austria), by centrifugation at 1000 g for 10 minutes at room temperature. Sera were frozen and stored at -80°C until testing.

### Spectral flow cytometry

Cryopreserved PBMCs were thawed and washed in RPMI before resuspension in buffer (0.2% FBS in PBS) at concentration of 1 x 10^6^ cells/ml in FACS tubes (Falcon^®^ Brand, VWR). Before addition of the cocktail of 20 monoclonal antibodies (**Supplementary Table 1**), cells were blocked with a cocktail of FcγR (1/15 dilution, Miltenyi), Novablock (Phitonex), and stained with live/dead violet viability dye, for 20 mins at 4°C. After washing, 30 μl of antibody cocktail was added to the cells, and the mix incubated for 30 minutes at 4°C. The stained PBMCs were washed and resuspended in 200 μl buffer and samples acquired using a Cytek Aurora (USA) spectral flow cytometer. Data was processed using OMIQ (California, USA).

### SARS-CoV-2 neutralizing antibodies

To measure neutralizing antibodies, against the SARS-CoV-2, we utilized the TECO^®^ SARS-CoV-2 Neutralization Antibody Assay (TECO medical AG, Sissach, Switzerland). The assay was performed according to the protocol provided by the manufacturer. Briefly, serum samples were diluted in sample buffer (1:10, 1:30, and 1:90) and incubated at 37°C for 30 minutes in a 96-well plate coated with ACE2 (provided in the kit). The plate was washed 3 times with diluted wash buffer (provided in the kit) and incubated with (S)-RBD–horseradish peroxidase conjugate for 15 minutes at 37°C. Finally, stop solution (provided in the kit) was added and optical density measured within 5 minutes at 450 nm (SpectraMax iD3 Molecular Devices, USA). The inhibition rate was calculated, and a positive value with the cutoff set at ≥20%.

### Data analysis and statistics

Data analysis and statistical calculations were performed with GraphPad Prism v9 (GraphPad Software, CA, USA). Differences among the study groups were analyzed with either unpaired, parametric T test with Welch’s correction, or Brown-Forsythe and Welch ANOVA tests, with no correction for multiple comparisons. In addition, bivariate analysis with Spearman’s correlation coefficient was performed. All differences with p values of <0.05 were considered statistically significant.

## Results

### Study outline and clinical parameters

**Figure 1:**
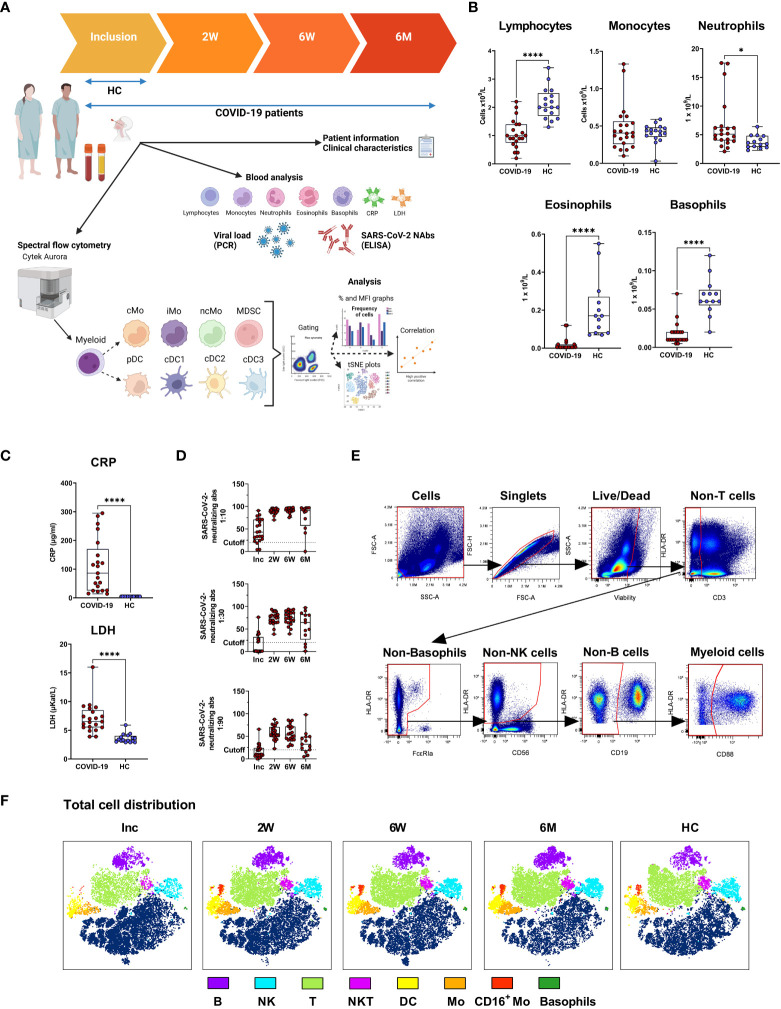
Study outline and experimental design. PBMCs and serum were collected from 21 COVID-19 patients and 16 healthy donors over a 6-7 month period. **(A)** Design of COVID cohort study illustrating timepoints for sample collection and experimental outputs, created with BioRender BioRender.com. Initial clinical assessments of **(B)** blood cell counts, **(C)** plasma C-reactive protein (CRP) and lactate dehydrogenase (LDH), and **(D)** neutralizing SARS-CoV-2 antibody titers. **(E)** Representative gating strategy to demonstrate live/dead gating and lineage exclusion, to leave myeloid cell populations for further analysis. **(F)** tSNE plots showing distinct clustering of all live cells, with 17 patients overlaid, and 16 controls overlaid. Data is represented as mean with 95% Cl, with significance of *p ≤ 0.05, ****p ≤ 0.0001, determined using Brown-Forsythe and Welch ANOVA tests. Inc, Study inclusion; 2W, 2 weeks; 6W, 6 weeks; 6M, 6-7 months; HC, healthy control. Mo, monocytes; cMo, classical; iMo, intermediate; ncMo, non-classical; MDSC, myeloid-derived suppressor cells; DC, dendritic cells; pDC, plasmacytoid; cDC1/2/3, conventional.

Patients suffering from COVID-19 and admitted to a hospital in Norrköping, Sweden were recruited into our study from May 2020 and throughout 2021, during and after the first and second waves of the pandemic in Sweden. Healthy controls who were confirmed SARS-CoV-2 negative by PCR and antibody tests, and had no prior history of COVID-19, were also recruited from within the hospital staff. Detailed clinical data were recorded for all individuals within the hospitalized cohort (**Table 1**). Whole blood, serum, and nasal swabs were collected at inclusion, and patients were followed up for further sample collection at 2 weeks, 6 weeks, and 6-7 months post-inclusion (**Figure 1A**). For this study, we have utilized longitudinal samples from 21 hospitalized COVID-19 patients and 16 healthy controls. Disease severity was defined according to the NIH guidelines, which are based largely on supplemental oxygen requirements (33). An initial assessment, at study inclusion, was made of an array of clinical parameters (**Figures 1B, C**), showing a significant decrease in lymphocytes, basophils, eosinophils, and neutrophils in the COVID-19 patients. The CRP and lactate dehydrogenase (LDH) levels were both significantly elevated in patients compared to healthy controls. All patients developed neutralizing SARS-CoV-2-specific antibodies that, in most individuals, lasted until the end of the study, even if the levels of neutralizing antibodies were significantly decreased at the 6-7 months timepoint compared to 2 and 6 weeks (**Figure 1D**). These findings are in agreement with previous publications (34–38). We performed correlation analysis of clinical parameters and found that CRP correlated with viral load at inclusion (**Supplementary Figure 1A**). Additional correlation analysis of clinical parameters and antibody levels revealed that anti-spike IgG levels and neutralizing antibody titers were negatively correlated with viral load at inclusion (**Supplementary Figure 1B**). The connection between viral load and both spike and nucleocapsid antibody levels has been shown previously (39, 40). Furthermore, there was negative correlation of neutralizing antibody titers and anti-spike IgG levels to CRP at inclusion (**Supplementary Figure 1C**). Following spectral flow data acquisition, lineage exclusion was performed to remove T cells, B cells, NK cells and basophils (**Figure 1E**, see **Supplementary Figure 2** for full gating strategies), and remaining cells were defined as myeloid and used for further analysis of monocyte and DC populations. Major immune cell types from within PBMCs, and their changing distributions over time were visualized following dimensionality reduction analysis (**Figure 1F**). At inclusion, there was a clear drop in many immune cell subsets, including T cells, B cells, NKT cells, monocytes, CD16^+^ monocytes, and DC, of which most recovered to normal levels during the study period.

### Increased levels of monocytes in the mononuclear myeloid compartment

**Figure 2:**
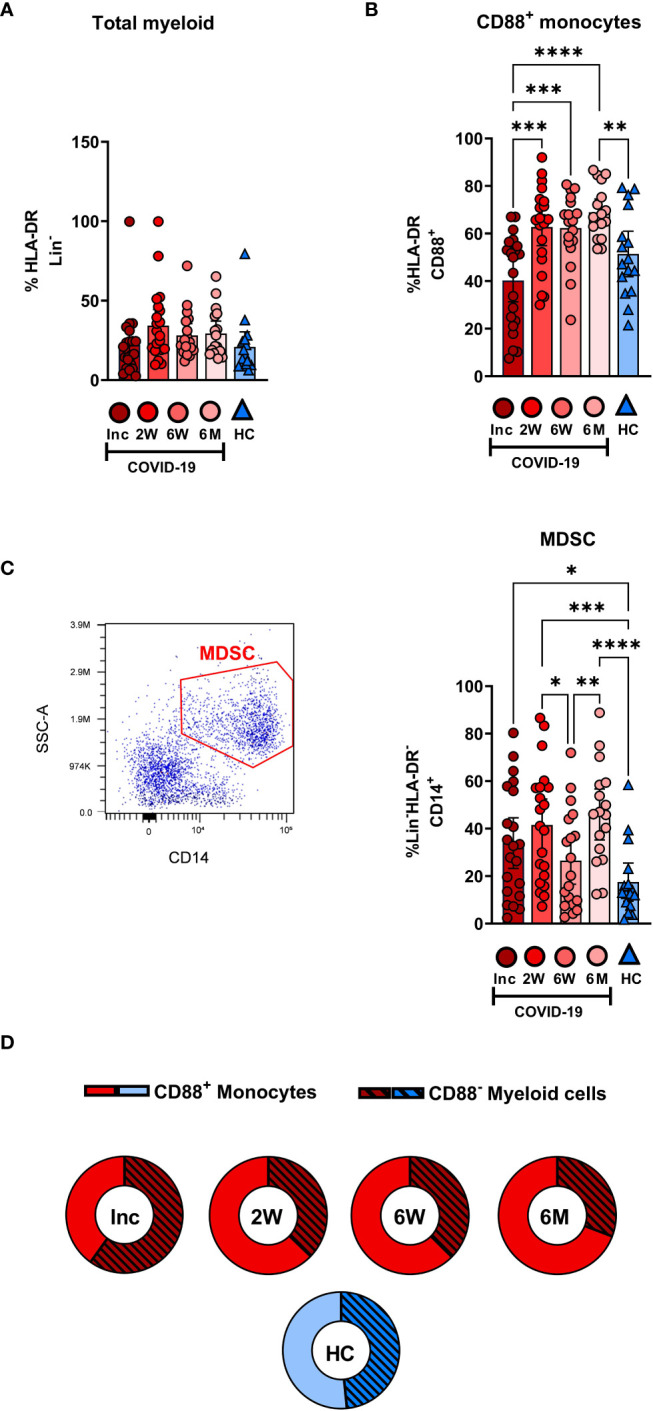
Dysregulation in the frequency of myeloid compartments persists for 6 months in hospitalized COVID-19 patients. PBMCs obtained from 21 COVID-19 patients and 16 healthy donors over a 6-7 month period were stained with monoclonal antibodies and assessed by flow cytometry to identify the myeloid cell subsets. Percentage of the cells comprising **(A)** the total HLA-DR^+^Lin^-^ myeloid compartment and **(B)** CD88^+^ monocytes. **(C)** Representative gating and percentage of CD14^+^HLA^-^DR-Lin^-^ MDSC. **(D)** Ratio of CD88^+^ monocytes to CD88^-^ myeloid cells. Data is represented as mean with 95% Cl, with significance of *p ≤ 0.05, **p ≤ 0.01, ***p ≤ 0.001, ****p ≤ 0.0001, determined using Brown-Forsythe and Welch ANOVA tests. Inc, Study inclusion; 2W, 2 weeks; 6W, 6 weeks; 6M, 6-7 months; HC, healthy control.

The proportion of total HLA-DR^+^ myeloid cells did not change significantly over the 6-7 months of the study (**Figure 2A**). Within the myeloid compartment in COVID-19 patients, monocytes were defined as CD88^+^ cells, with CD88 being exclusively expressed on blood monocytes (41). Monocytes increased significantly during the first 2 weeks after inclusion and reached a plateau that was significantly higher at 6-7 months post COVID-19 compared to controls (**Figure 2B**). The proportion of CD14^+^HLA-DR^-^ MDSC was raised compared to healthy controls at inclusion and at 2 weeks and 6-7 months follow up, but surprisingly, there was a significant dip at the 6 week time point (**Figure 2C**). Compared to the healthy controls there was a shift towards the non-monocytes, i.e., CD88^-^ cells, in the myeloid compartment following COVID-19 (**Figure 2D**). Together our results show that there is a sustained shift in the balance of cell types within the myeloid compartment (**Figure 2D**), despite the total level of myeloid cells remaining the same (**Figure 2A**).

### Long term changes in classical and intermediate monocytes post COVID-19

**Figure 3:**
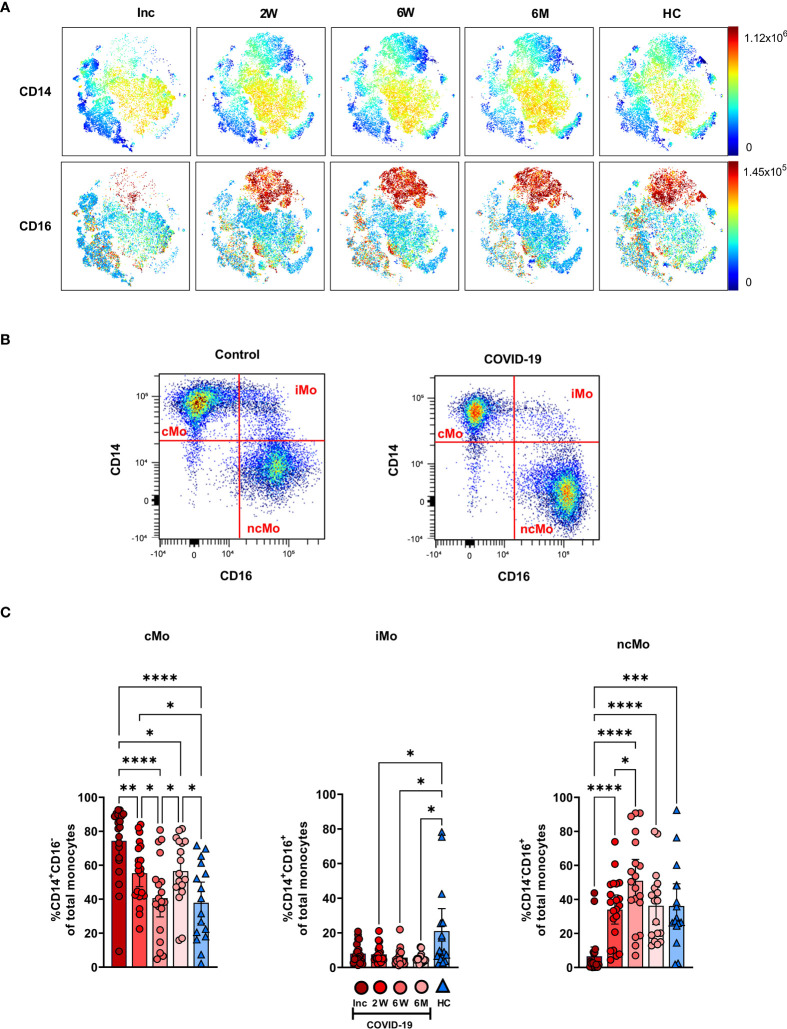
Classical and intermediate monocyte cells subsets remain altered in COVID-19 patients at 6 months. PBMCs were collected from COVID-19 patients that required hospitalization (N=21) and healthy controls (N=16) over a 6−7-month period. Cells were stained with antibodies for flow cytometry to define the monocyte subsets. **(A)** tSNE plots displaying myeloid cell clusters, colored according to their intensity of CD14 and CD16 expression. **(B)** Representative plots illustrating how monocyte subsets were defined. **(C)** Frequencies of the CD14^+^CD16^-^ cMo, CD14^+^CD16^+^ iMo, and CD14^-^CD16^-^ ncMo. Data is represented as mean with 95% Cl, with significance of *p ≤ 0.05, **p ≤ 0.01, ***p ≤ 0.001, ****p ≤ 0.0001, determined using Brown-Forsythe and Welch ANOVA tests. Inc, Study inclusion; 2W, 2 weeks; 6W, 6 weeks; 6M, 6-7 months; HC, healthy control.

The CD14^+^CD16^-^ cMo, CD14^+^CD16^+^ iMo and CD14^-/lo^CD16^+^ ncMo were examined to compare COVID-19 patients to healthy controls (**Figure 3**). The CD14^+^ and CD16^+^ cell levels within the myeloid compartment were initially low and increased over the 6-7 months (**Figure 3A**). Following gating on monocyte subsets (**Figure 3B**), the proportion of cMo at inclusion was significantly higher than in healthy controls and remained significantly elevated even after 6-7 months (**Figure 3C**). The iMo were reduced for the entire duration of the study (**Figure 3C**). A drastic reduction in ncMo at inclusion was observed in patients, but had already returned by 2 weeks to a level comparable to controls (**Figure 3C**). Together these data revealed that there was a sustained effect on the proportion of cMo and iMo in COVID-19 patients, while the change in ncMo was restored within 2 weeks.

### Circulating conventional DC subsets and plasmacytoid DC levels recovered after initial depletion

**Figure 4:**
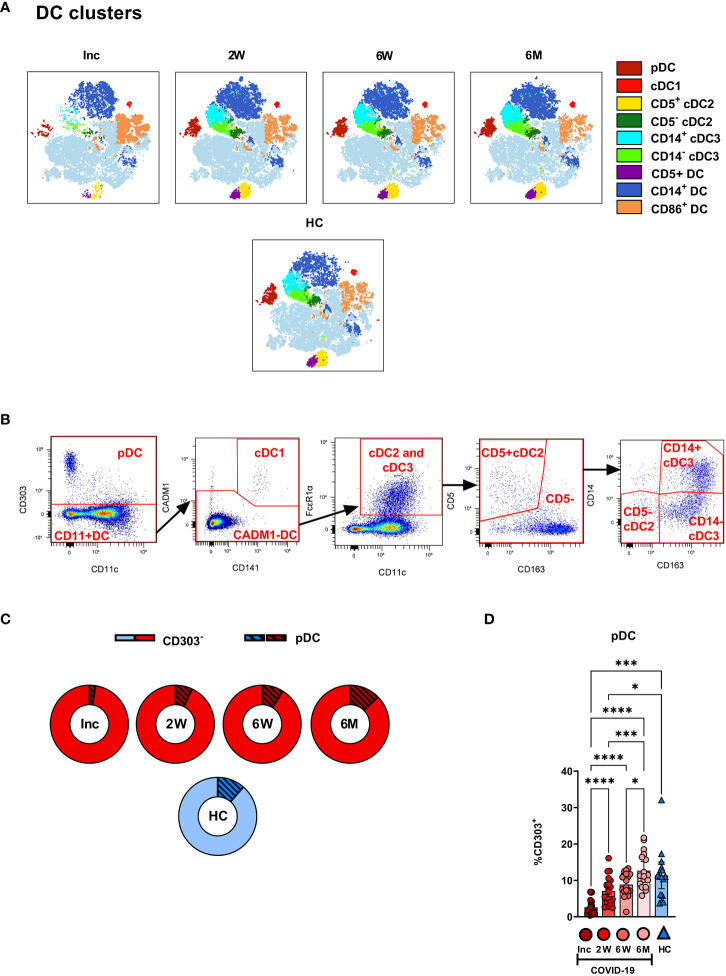
Plasmacytoid DCs in COVID-19 patients are severely depleted in blood during COVID-19. PBMCs collected from hospitalized COVID-19 patients (N = 21) and healthy controls (N = 16), were stained for flow cytometry to observe the effect of SARS-CoV-2 on the overall DC compartment. **(A)** tSNE plots illustrating the changing distribution of DC within CD88^-^ myeloid cells. **(B)** Gating strategy for DC subsets. **(C)** Ratio of CD303^-^ cells to pDC during COVID-19. **(D)** Percentages CD303^+^ pDC. Data are presented as mean with 95% Cl, with significance of *p ≤ 0.05, ***p ≤ 0.001, **** p ≤ 0.0001, determined using Brown-Forsythe and Welch ANOVA tests. Inc, Study inclusion; 2W, 2 weeks; 6W, 6 weeks; 6M, 6-7 months; HC, healthy control.

**Figure 5:**
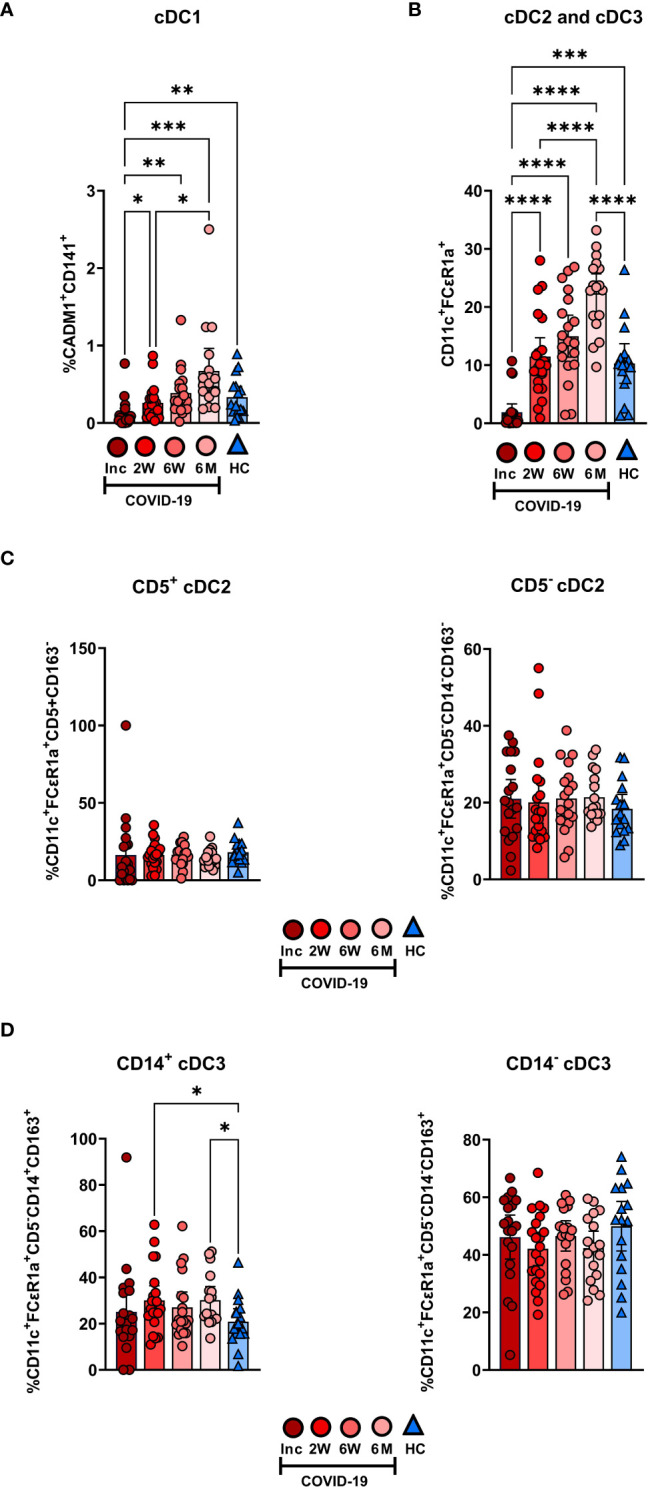
Initial depletion of classical DC populations during COVID-19. PBMCs from hospitalized COVID-19 patients (N = 21) and healthy donors (N = 16), were stained for flow cytometry to observe changes in the composition of the cDC compartment during COVID-19. Proportions of **(A)** CADM1^+^CD141^+^ cDC1, **(B)** CD11c^+^FCϵR1a^+^ cDC2 and cDC3 combined, **(C)** CD5^+^CD163^-^ cDC2 and CD5^-^CD163^-^ cDC2, and **(D)** CD5^-^CD14^+^CD163^+^ cDC3 and CD5^-^CD14^+^CD163^+^ cDC3 are shown. Data is presented as mean with 95% Cl, with significance of *p ≤ 0.05, **p ≤ 0.01, ***p ≤ 0.001, ****p ≤ 0.0001, determined using Brown-Forsythe and Welch ANOVA tests. Inc, Study inclusion; 2W, 2 weeks; 6W, 6 weeks; 6M, 6-7 months; HC, healthy control.

We assessed the different circulating DC subsets (**Figures 4A, B**) and found decreased levels of all DC subsets in the patients at study inclusion. The proportion of pDC were significantly reduced in COVID-19 patients at inclusion and that these cells started to recover already at two weeks (**Figure 4A**). The pDC fraction amongst the CD88^-^ myeloid cell compartment clearly increased until it reached a similar level to healthy controls (**Figure 4C**). The level had increased significantly by 2 weeks but did not fully return to normal until 6 weeks (**Figures 4A, C, D**). Next, we explored the different cDC subsets in COVID-19 patients. Among the cDC, CD141^+^ cDC1 constitute a very small fraction (20). This DC subset (**Figure 4A**) was significantly reduced at inclusion but throughout the 6-7 months studied, returned to a level comparable to controls (**Figure 5A**). Here, we analyzed cDC2 and cDC3 as one combined population due to the contrasting methods used by different groups to further define distinct cDC subpopulations within classical cDC2. While the proportion of the cumulative cDC2 and cDC3 subset was significantly reduced at inclusion, this recovered over the study period and even significantly surpassed the levels found in healthy controls and at 6-7 months (**Figure 5B**). Following this, cDC2 and cDC3 were further divided based on their expression of CD5 and CD14, respectively. Interestingly, neither the CD5^+^ cDC2 nor the CD5*^-^* cDC2 were significantly altered at any of the study timepoints (**Figure 5C**). There was a significant increase in CD14^+^ cDC3 at the 2 week and 6-7 month time points, but no change in the CD14^-^ cDC3 (**Figure 5D**). Together, we found that both pDC and cDC1 recovered from initial reductions during the study. Additionally, there were major alterations over time in the combined cDC2 and cDC3 population, though the proportions of the subpopulations within these DC subsets were unaffected.

### Prolonged decrease in PD-L1 and elevated CD86 and HLA-DR expression levels on monocyte subsets

**Figure 6:**
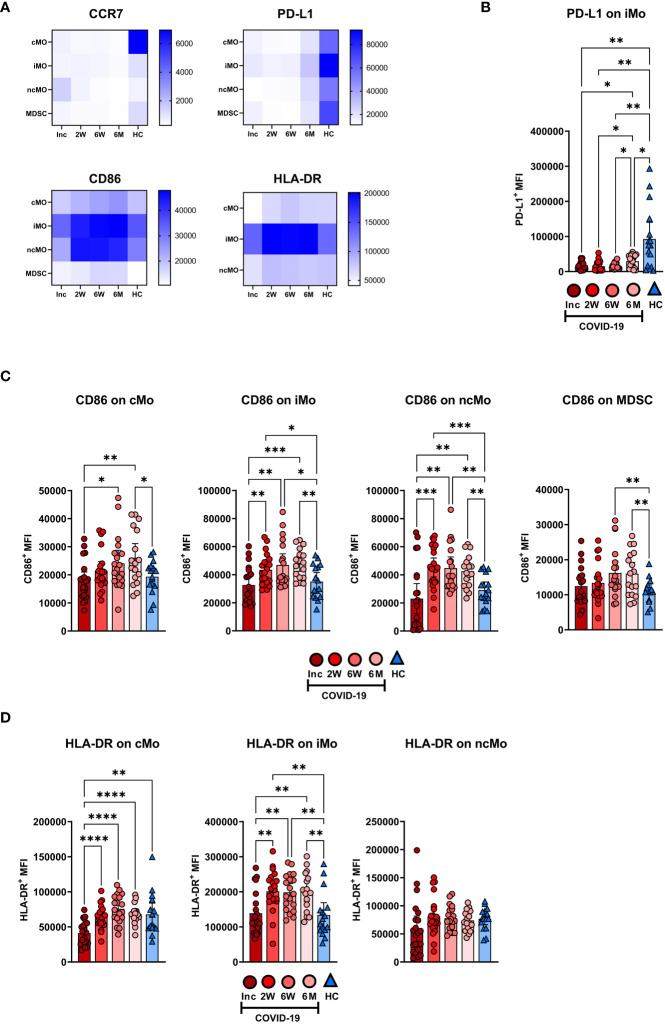
Long-term changes in the phenotype of blood monocyte subsets and myeloid derived suppressor cells in COVID-19 patients. PBMCs were collected from hospitalized COVID-19 patients (N=21) and healthy controls (N = 16) over a 6−7 month period. **(A)** Heatmaps showing the mean fluorescence intensity (MFI) of CCR7, PD-L1, CD86, and HLA-DR on monocyte subsets and MDSC, from all donors. **(B)** Representative graph of PD-L1 on iMo. MFI of **(C)** CD86 and **(D)** HLA-DR on monocyte subsets and MDSC. Data is represented as mean with 95% Cl, with significance of *p ≤ 0.05, **p ≤ 0.01, ***p ≤ 0.001, ****p ≤ 0.0001., determined using Brown-Forsythe and Welch ANOVA tests. Inc, Study inclusion; 2W, 2 weeks; 6W, 6 weeks; 6M, 6-7 months; HC, healthy control.

The expression levels of functional surface markers were examined on monocyte subsets and MDSC (**Figure 6A**; **Supplementary Figure 3**). The clearest pattern was the decreased PD-L1 expression, which was observed at all time points on the monocyte subsets and the MDSC (**Figures 6A, B**; **Supplementary Figure 3**). In addition, there were increased CD86 and HLA-DR levels from the 2 week timepoint onwards on the monocyte subsets (**Figures 6A, C, D**). The iMo were selected to represent the PD-L1 expression pattern seen across all monocytic cell types, which was significantly reduced at all time points compared to healthy controls (**Figure 6B**). A detailed look at CD86 expression levels revealed a significant increased expression from 2 weeks onwards in iMo and ncMo, from 6 weeks onwards in MDSC, but not until 6 months in cMo (**Figure 6C**). HLA-DR expression was initially reduced in cMo before reverting to levels found in healthy controls at the 2 week time point. At the same time, there were increased HLA-DR levels from the 2 week time point onwards in iMo. HLA-DR expression on ncMo was similar at all time points (**Figure 6D**).

### Prolonged decrease in PD-L1 expression on all DC subsets and elevated CD86 levels on conventional DC subsets

**Figure 7:**
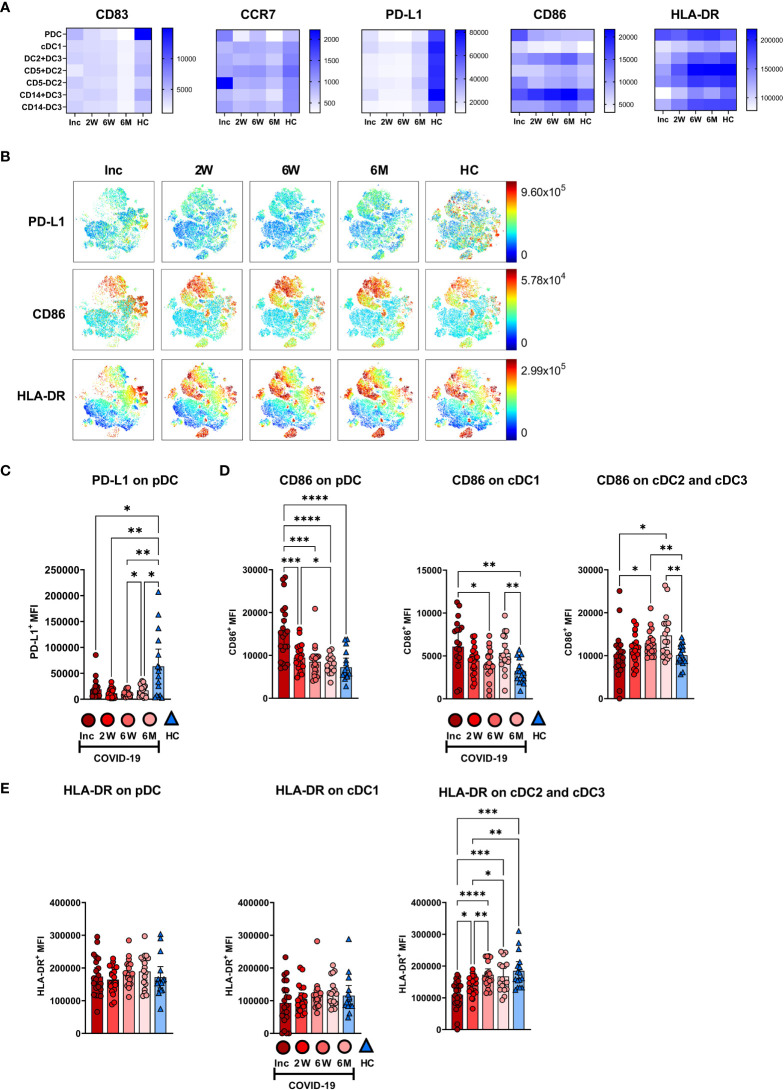
Altered phenotype of circulating dendritic cell subsets in COVID-19 patients. PBMCs collected from hospitalized COVID-19 patients (N = 21) and healthy controls (N = 16) over a 6−7 months period, were assessed for phenotypical changes. **(A)** Heatmaps showing the mean fluorescence intensity (MFI) of CD83, CCR7, PD-L1, CD86, and HLA-DR on different DC subsets. **(B)** tSNE plots showing CD88^-^ myeloid cells, colored according to intensity of PD-L1, CD86, and HLA-DR expression. **(C)** Representative graph of PD-L1 on pDC. MFI of **(D)** CD86 and **(E)** HLA-DR on DC subsets. Data is represented as mean with 95% Cl, with significance of *p ≤ 0.05, **p ≤ 0.01, ***p ≤ 0.001, ****p ≤ 0.0001., determined using Brown-Forsythe and Welch ANOVA tests. Inc, Study inclusion; 2W, 2 weeks; 6W, 6 weeks; 6M, 6-7 months; HC, healthy control.

The DC subsets were assessed for the expression levels of functional surface markers (**Figures 7A, B**; **Supplementary Figure 4**). There was a clear reduction in CD83 at 6 months and a decrease in PD-L1 at all time points, across all DC subsets (**Figure 7A**). The pDC and cDC3 were found to have decreased expression of CCR7 at the 6-7 month time point (**Figure 7A**; **Supplementary Figure 4**). PD-L1 was significantly decreased during the entire study across all DC subtypes studied (**Figures 7A, C**; **Supplementary Figure 4**). CD86 expression on pDC was significantly increased at inclusion but returned, by 2 weeks, to a comparable level to controls and remained so for the remainder of the 6-7 months post-COVID-19. On cDC1, CD86 was increased at both inclusion and the 6-7 month time point, but not in the intervening period. In the cumulative cDC2 and cDC3 population, CD86 expression increased to a significantly higher level than controls at 6 weeks and 6-7 months (**Figure 7D**). Only cumulative cDC2 and cDC3 showed any significant changes to HLA-DR expression, with a significant increase over time, until the expression from 6 weeks onwards showed no significant difference to healthy controls (**Figure 7E**). Taken together these data show that COVID-19 elicited lasting alterations in PD-L1 and CD86 expression levels on DC subsets.

### CRP at inclusion correlated with alterations in monocyte and DC subsets in COVID-19 patients

**Figure 8:**
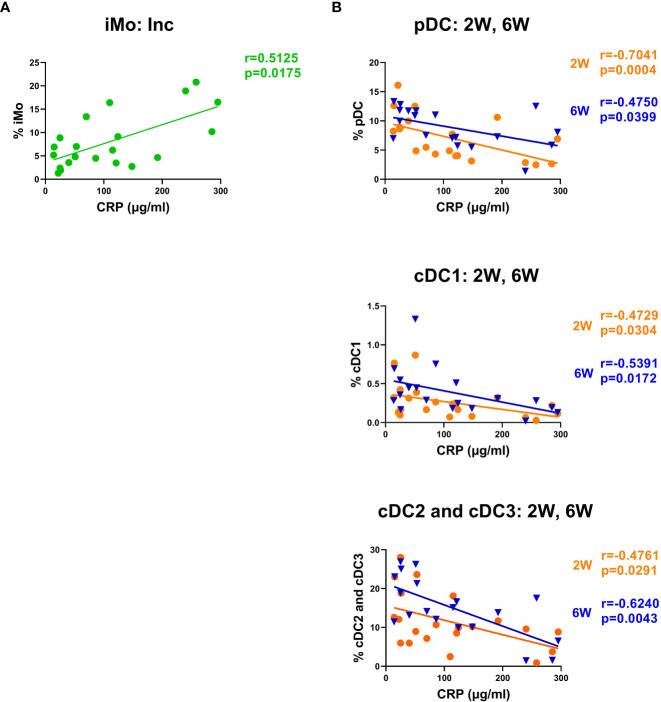
CRP levels correlated negatively with intermediate monocyte levels and with the recovery of DC subsets. Bivariate analysis with Spearman’s correlation coefficient was performed using an array of clinical parameters and myeloid cell types. Significant correlations shown for **(A)** iMo at inclusion and **(B)** circulating DC subsets at 2 and 6 weeks post-inclusion, all against CRP. The p value and Spearman’s correlation coefficient (R) are shown for each analysis. N = 21. Inc, Study inclusion; 2W, 2 weeks; 6W, 6 weeks; 6M, 6-7 months; HC, healthy control.

To determine if the initial inflammatory response to the SARS-CoV-2 infection affected the monocyte and DC subsets in the COVID-19 patients, we assessed correlations between myeloid cell populations and clinical parameters such as age, biological sex, and laboratory blood analysis such as viral load, LDH, and CRP. There were no significant differences between age or biological sex in the different myeloid populations. We found that the proportion of iMo at inclusion positively correlated with CRP (**Figure 8A**), and negative correlations were found with CRP and all DC subtypes, both at 2 and 6 weeks post-inclusion (**Figure 8B**).

## Discussion

To address the lack of knowledge concerning myeloid cells in COVID-19 we have investigated the effects that SARS-CoV-2 infection exerts, both initially and long-term, on monocyte and DC subsets. We found both initial and long-term alterations to the frequencies and phenotypes of these cells, indicating an immune dysregulation and dysfunction.

Monocytes are today divided into three functionally different subpopulations distinguished primarily by their expression of CD14 and CD16 (9). In mouse models of acute lung injury and in acute respiratory distress syndrome, as seen in severe COVID-19, circulating monocytes have been shown to play a pivotal role in driving inflammation (42). Monocyte frequencies have been found to be lower during acute COVID-19 compared to convalescence (43). In line with this, the levels of overall monocytes in the patients in our study were significantly decreased at inclusion, when compared to the later time points. A factor affecting the levels of monocyte subsets could be virus-mediated cell death during COVID-19, since they all express the SARS-CoV-2 receptor, ACE2 (43), and there is evidence of viral antigens present within ncMo for over a year after initial infection (44). In addition, CD16^+^ monocytes can also be infected in an ACE2-independent manner, leading to inflammatory cell death (45). Our observed sustained increase in cMo in COVID-19 patients in comparison to healthy controls is consistent with previously published data (26). The elevated levels of these cells at 6-7 months could be part of the host resolving long-term effects of the SARS-CoV-2 infection as the key roles of cMo include tissue repair and anti-apoptotic functions (46, 47). We saw an initial drastic drop in the level of ncMo, which recovered in convalescence, consistent with other reports (26, 48). This initial drop could be due to the recruitment of CD16^+^ myeloid cells to inflamed lung tissue during infection (27, 48, 49). Different patterns have been seen in severe compared to mild COVID-19 in the levels of circulating iMo, with decreased levels in severe disease when measured during active disease (27). In our patient cohort we found decreased levels of iMo compared to healthy controls, which failed to recover, indicating a long-lasting effect.

Recent studies, through single-cell resolution methods, point to the expansion of suppressive myeloid cells in the blood as a hallmark of severe COVID-19 (17, 26), as confirmed by our data. These cells (MDSC) are a heterogeneous population of myeloid cells that exist during normal conditions in tissues (50). The levels of MDSC have potent immune suppressive properties and have been found to correlate to impaired T cell responses (51, 52). This sustained elevation suggests a long-lasting suppressive state, together with the suppressive T cell phenotypes defined in our COVID-19 cohort (3) indicate a long lasting impaired immune response after COVID-19.

In line with other studies, we found that COVID-19 had a major impact on circulating DC subpopulations (26), with major reductions during active disease for pDC (17), cDC1 (27), and combined cDC2/cDC3 (29). The reduction in DC subsets at inclusion could be due to several factors such as redirection of circulating pDC and cDC1 to lymphoid tissue (28, 53), and the recruitment of cDC2 subtypes into the lung tissue, though this is not seen for pDC and cDC1 (27, 54). In addition to relocation from circulation to tissue, the decrease in DC might depend on their destruction by direct or indirect viral effects, since SARS-CoV-2 can bind and enter DC through CD147 and DC-SIGN (55, 56). The combined cDC2/cDC3 population increased to levels higher than in healthy controls and these elevated levels persisted long-term. A lasting effect with higher levels of HLA-DR^+^CD11c^+^ DC has been noted in individuals needing hospitalization, whereas normal levels of CD141^+^ DC, i.e., cDC1 were observed (32). This elevated level of cDC2/cDC3 could play a role in ongoing, long-term, systemic inflammation due to the damage inflicted by COVID-19 on the host. Almost all DC functions are affected during COVID-19 (28) with probable consequences for the progression and outcome of the illness. Initial type I and III IFN responses are essential to the resolution of viral infection and are found to be impaired in severe COVID-19 (57), which could be due to initial DC depletion. Given the importance of DC for the initial activation of T cell responses, their depletion or tissue relocation could hamper the subsequent T cell response (58). Overall, the long-lasting alteration in the DC compartment could explain the sustained T cell dysfunction seen following COVID-19 (3, 59).

In different diseases such as cancer and severe infections, the phenotype and functionality of circulating monocyte and DC subsets are altered (60, 61). One of the immune checkpoint molecules affected is CD86, which is known to be upregulated in settings with ongoing immune activation and chronic inflammation (62–64). Our phenotypic analysis showed little to no decrease in CD86 expression during acute disease, on monocyte subsets and MDSC, and this was followed by elevated CD86 levels on all these cells at 6-7 months. Previous studies have shown that monocyte subsets, and especially iMo, have decreased CD86 during acute infection (35, 65). We found an increased level of expression on pDC and cDC1 at inclusion but not cDC2/cDC3. The elevated CD86 levels on pDC have been documented during acute SARS-CoV-2 infection (66). However, other studies found no effect on pDC, or on the myeloid DC subsets in acute disease (4, 10, 29, 67). The expression of CD86 returned to levels comparable to healthy controls quickly for pDC but remained elevated on cDC1, cDC2, and cDC3 subsets at 6-7 months. This contrasts with a previously published study, which showed long-term reduction in CD86 expression on pDC and HLA-DR^+^CD11c^+^ DC subsets in hospitalized COVID-19 patients (32). Elevated CD86 expression was found on ki67^+^ cDC2 and cDC3 subsets, i.e., a similar pattern as we have seen for these DC subsets, highlighting that the level seems to be connected to the time that they have been in circulation (29).

We suspect that the ongoing, low-level, systemic inflammation from lung repair following COVID-19 (68) could be the cause of the increased CD86 on monocyte and DC populations since CD86 expression is elevated in chronic HIV-1 and HCV, both infections that cause inflammation (62–64). The elevated CD86 expression we found on the myeloid cells could play a role in hyperactivation or T cell impairment. The differences between our and other results could be due to the method of defining DC subsets, with many studies assessing CD14^-^HLA-DR^+^CD11c^+^ DC and not the different cDC subsets we have used in our study.

Previous studies have shown decreased HLA-DR levels in acute COVID-19 for cMo and ncMo (17, 35, 48, 67, 69), which is in accordance to our results. The iMo have been found to have downregulated HLA-DR during severe acute COVID-19 (17, 48, 69), which differs to our observation of no effect on HLA-DR at inclusion. Regarding the DC subsets, the HLA-DR expression in severe COVID-19 has previously been found to be reduced across all circulating DC besides cDC1 (26). In our study HLA-DR was only significantly altered on cDC2/3 where it was reduced at early time points before returning to levels found in controls, which is in agreement with Marongiu et al. who showed a reduction of HLA-DR in cDC2 and cDC3 subsets (70). The observed initial low expression of HLA-DR on cDC1, cDC2/cDC3, cMo, and ncMo (29, 69, 71, 72) could be a sign of impaired functionality and part of the immunosuppression seen in severe disease.

PD-L1 has been shown to be dysregulated in COVID-19 patients (73). In our study we observed a consistently lower PD-L1 surface expression across all myeloid cells. For pDC and cDC subsets this aligns with gene and protein expression levels in hospitalized patients during acute disease (32, 73, 74). In contrast, an increase in PD-L1 expression has been seen in other studies on bulk monocytes (67, 75), and circulating DC subsets (66, 67). In our data this reduced expression of PD-L1 persisted for the entire duration of the study, as was seen also in a previous study at 7 months post-COVID-19 (32). The loss of PD-L1 on monocyte and DC subsets might be due to shedding of soluble PD-L1, which is found to be elevated in the serum of COVID-19 patients (73) and to be one feature of critical COVID-19 (76). The lasting reduction that we observe in PD-L1 expression across all myeloid cell subsets requires further study to explore if it plays any role in COVID-19 pathogenesis and if it is due to these cells shedding PD-L1.

Concerning the migratory receptor CCR7, there was little change in the DC and monocyte subsets during acute disease compared to healthy controls, while long-term effects included decreased CCR7 on cMo, pDC and the two cDC3 populations. This decrease is in accordance with previous findings for cDC at 7 months (32), but not for pDC, which had long-term increased CCR7 (32). We did not find any major alterations in the surface expression of CD83 across DC, which concurs with Venet et al. (74).

Overall, we identified marked alterations to the expression of surface markers across myeloid cell types following COVID-19, which could play a part in the resulting immune suppression. The long-term changes to the surface expression of these proteins on monocytes and DC may be due to ongoing inflammation or epigenetic changes resulting from severe COVID-19 (77), possibly altering progenitors in the bone marrow.

There are many reports that demonstrate that there is higher morbidity and mortality in males than females. Sex hormones have been implicated in the ability of females to cope with the infection better. In addition, factors such as the innate response, cytokines, T cells, and monocytes differ between males and females (78–80). Takahashi et al. found that male patients had higher levels of ncMo than female during acute disease (79). We could not find this difference in ncMo, or any other differences in the myeloid compartment, between males and females.

COVID-19 severity has, besides age and biological sex, been linked to an array of clinical parameters such as the level of soluble urokinase plasminogen activator receptor (suPAR), CRP, LDH, and viral load (81–86). When exploring correlations between the clinical parameters measured in this study, the viral load correlated positively with the CRP levels and negatively with anti-spike IgG and neutralizing SARS-CoV-2 antibody levels, at inclusion. In addition, the COVID-19 patients with high CRP levels had lower levels of anti-spike IgG and neutralizing antibodies at inclusion. We have previously shown that viral load correlates to disease severity (87), that is in turn connected to suboptimal development of germinal centers (88) that in turn might be the reason for the delayed IgG response. However, our correlations do not definitely mean causality and so their underlying mechanisms warrant further investigation.

This negative association between CRP and antibody levels early on during SARS-CoV-2 infection has, to our knowledge, not yet been made. It has previously been shown that higher levels of SARS-CoV2 specific antibodies during convalescence correlated with initial higher CRP levels (89), though we did not see this.

During acute disease in hospitalized patients, the levels of pDC, cDC2, and CD163^+^CD14^-^ cDC3 are negatively correlated to CRP levels (29, 69). We confirm this to be the case for all circulating DC subsets at acute and six weeks post-inclusion, i.e., a faster DC recovery was predicted by lower initial levels of CRP. This negative effect on DC levels could be due to CRP impairment of DC development (90) and the slow recovery of DCs could affect the ability to respond to new infections and activate T cell responses. The levels of ncMo and iMo have been previously found to negatively correlate to CRP in acute disease (91), while we found a positive correlation for iMo levels in our study. Raised CRP is part of a highly inflammatory environment in COVID-19 with prolonged high viral load due to the lack of a strong type I IFN response (92). This in turn might have long-term effects on monocyte and DC subsets, which our data strongly supports.

A potential limitation of our study is the imperfect age and sex matching of the controls to the patients. This is of particular importance for some DC subsets such as pDC, which are decreased in older (>40 years) healthy individuals, whereas there are no significant effects on cDC (93, 94). Given that the levels of pDC returned to the levels of healthy controls we do not believe this to be an issue. While biological sex does not seem to play a role for the levels of MDSC in general, their levels could be influenced during disease. A study of MDSC in mild to severe COVID-19 found higher levels of monocytic MDSC in males than in females (80). The increase we found of MDSC in blood from hospitalized COVID-19 patients did not correlate with biological sex or age, however, our cohort was relatively small and did not contain cases of mild disease, so a larger cohort may be needed to observe these associations. Another effect of our relatively small cohort is that with large number of parameters explored we have comparatively low statistical power. The MDSC levels increase with age and highly elevated levels are seen in severe infections and cancers (95–101). A general problem when comparing data from different COVID-19 studies is the definition of disease severity, that differs depending on country. We defined severity according to the NIH guidelines, which are based largely on supplemental oxygen requirements (33), as opposed to the WHO scale (102).

In conclusion, given the long-lasting changes in the monocyte, DC and MDSC compartments, as seen in the altered frequencies of cell populations and expression levels of various surface markers, it is evident that COVID-19 impacts the development and functionality of these cells. Further studies will be required to determine for exactly how long these alterations persist after severe COVID-19, and if they affect the type and quality of immune responses elicited against future infections.

## Data availability statement

The raw data supporting the conclusions of this article will be made available by the authors, without undue reservation.

## Ethics statement

The studies involving human participants were reviewed and approved by Swedish Ethical Review Authority. The patients/participants provided their written informed consent to participate in this study.

## Author contributions

FH, MG, CS, JN, and HW conducted experiments. FH, MG, CS, JN, HW, SN, and ML analyzed the data. FH, MG, and ML were involved in the writing of the initial manuscript, and FH, MG, CS, JN, AN-A, AH, MH, JS, SN, and ML helped in the revision of the manuscript. ML designed the experiments. AN-A, AH, MH, JS, SN, and ML were involved in establishing the cohort study. All authors read and approved the final manuscript.
